# Effect of ketamine combined with lidocaine in pediatric anesthesia

**DOI:** 10.1002/jcla.23115

**Published:** 2019-11-15

**Authors:** Hua Fang, Hua‐Feng Li, Miao Yang, Fang‐Xiang Zhang, Ren Liao, Ru‐Rong Wang, Quan‐Yun Wang, Peng‐Cheng Zheng, Jian‐Ping Zhang

**Affiliations:** ^1^ Department of Anesthesiology Guizhou Provincial People's Hospital Guiyang China; ^2^ Department of Anesthesiology Guizhou University People's Hospital Guiyang China; ^3^ Department of Anesthesiology West China Second University Hospital Sichuan University Chengdu China; ^4^ Department of Anesthesiology West China Hospital Sichuan University Chengdu China; ^5^ Guizhou University Research Center for Analysis of Drugs and Metabolites Guizhou University Guiyang China

**Keywords:** injectable anesthesia, ketamine, lidocaine, pediatric anesthesia

## Abstract

**Background:**

We conducted a randomized clinical trial to determine whether adjunctive lidocaine diminishes the incidence of adverse effects in pediatric patients sedated with ketamine.

**Methods:**

This case‐control study involved 586 consecutive pediatric patients necessitating anesthesia. Then systolic blood pressure, heart rate, respiratory rate, and blood oxygen saturation were observed. Alanine aminotransferase (ALT), aspartate aminotransferase (AST), urea nitrogen (BUN), and creatinine (Cr) levels were tested. General dose of ketamine, the time of onset and duration of anesthesia and postoperative recovery, anesthesia effect, and adverse reaction were subsequently compared. High‐performance liquid chromatography was employed to detect ketamine concentration at different time points after administration, and the postoperative cognition function was further evaluated.

**Results:**

Intra‐ and post‐operation, the rising degree of ALT, AST, BUN, and Cr in patients treated with ketamine was higher than those in patients treated with the ketamine‐lidocaine complex. General dose of ketamine, the time of onset and duration of anesthesia, postoperative recovery time, and the incidence rate of adverse reaction in patients treated with ketamine‐lidocaine complex were lower, but the concentration of ketamine was higher compared to the patients treated with ketamine. In patients treated with the ketamine‐lidocaine complex, elimination half‐life of ketamine was prolonged, the area under curve was increased, and the plasma clearance rate was decreased relative to those with ketamine alone.

**Conclusions:**

Ketamine combined with lidocaine may be beneficial in shortening the onset of anesthesia, promoting postoperative awake, prolonging elimination half‐life, increasing area under curve, and decreasing plasma clearance rate and incidence of adverse reactions.

## 1 INTRODUCTION

Adequate pain control plays an important role in patients undergoing operation and painful procedures, which is particularly essential for pediatric patients undergoing operation. During operation process, inadequate pain control may lead to incomplete procedure and adverse complications.^1^ To facilitate a higher success rate, a majority of procedures are performed under anesthetic effect, especially for uncooperative circumstances among children.^2^, ^3^ Therefore, administering an appropriate degree of anesthesia to cope with the procedural demands remains a challenge for the anesthesiologist. Recently, administering a combination of two or more agents has become a readily adopted solution to meet the procedural requirements.^4^

Ketamine is acknowledged among the oldest hypnotic agents for anesthesia due to its analgesic properties and minimal suppressive effects on respiration.^5^ Based on its effective bronchodilating respiratory properties and stable hemodynamics, ketamine use has been reported in patients with brain injury, resulting in increased clinical use.^6^ Moreover, the administration of ketamine as an anesthetic has branched for extraperitoneal procedures, cardiac catheterization, orthopedics, and skin grafting, as well as otolaryngology and optical procedures.^7^ Lidocaine, firstly synthesized by the Swedish chemist Lofgren, has been used extensively, such as for pain management, clinical anesthesia, nervous system diseases, arrhythmia, treatment of respiratory diseases, and gastrointestinal diseases.^8^ The administration of a high concentration of lidocaine led to the illustration of adverse reactions such as skeletal muscle twitching, slurred speech, and drowsiness.^9^ As an amide local anesthetic, the analgesic, prokinetic, and antiarrhythmic properties of lidocaine are evident upon systemic administration in humans.^10^ A previous study conducted by Kvarnström et al^11^ revealed the analgesic effects of lidocaine on peripheral neuropathic pain. Besides, intravenous lidocaine before operation exhibited its preventive effect on propofol‐induced injection pain and hyperalgesia, and subsequently protected against bronchial reactivity due to bronchotracheal relaxation during the operation, consequently resulting in the increase in depth of general anesthesia.^12^ A double‐blind and randomized study performed by Jendoubi et al^13^ flagged the use of intravenous lidocaine and ketamine as safe and effective adjuvants to decrease opioid consumption and for early pain management after open nephrectomy procedure. Moreover, pretreatment with lidocaine (20 mg) and ketamine (5 mg) with venous occlusion for 1 minute is the most effective in attenuating the pain on propofol injection vs pretreatment with the lidocaine combined with metoclopramide or plain lidocaine.^14^ However, currently, there is an inadequacy in clinical trials to provide evidence about the efficacy and safety of ketamine and lidocaine in combination on pediatric anesthesia. Hence, we hypothesized that a combination of lidocaine and ketamine would produce a clinically relevant decrease in the adverse effects in pediatric patients.

## 2 METHODS

### 2.1 Ethics statement

The present study was conducted under the approval of the medical ethics committee of the Guizhou Provincial People's Hospital and Guizhou University People's Hospital. The participants and patient guardians included in the current study signed written informed consents.

### 2.2 Study subjects

The study recruited children (n = 586) who were operated upon in the Guizhou Provincial People's Hospital and Guizhou University People's Hospital from a period between March 2013 and December 2015, including 338 cases undergoing surgical intervention for hernia, 140 cases undergoing cleft lip and palate repair, and 108 cases undergoing appendectomy. The operation time period ranged between 15 and 100 minutes with a mean time of 55.26 ± 4.45 minutes. Among the participants, there were 478 males and 108 females aged 2‐12 years (mean age of 6.35 ± 2.32 years) and weighing 9.13‐55.42 kg (mean weight of 22.03 ± 6.28 kg). Prior to operation, the pediatric patients underwent physical examination with liver and kidney function testing as well as blood and urine testing to ensure the parameters were all within the normal range. The pediatric patients were then randomly divided into the observation group and the control group (n = 293, respectively). The observation group was comprised of 170 pediatric patients undergoing hernia repair, 71 undergoing cleft lip and palate repair, and 52 undergoing appendectomy; 237 males and 56 females, aging between 1 and 12 years (mean age of 6.91 ± 2.23 years) and weighing 8.11‐45.17 kg (mean weight of 25.93 ± 6.92 kg), respectively. The control group consisted of 168 cases undergoing hernia repair, 69 cases undergoing cleft lip and palate repair, and 56 cases undergoing appendectomy. Among them, there were 241 males and 52 females, aging between 1 and 13 years (mean age of 6.63 ± 2.17 years) and weighing 8.04‐42.48 kg (mean weight of 25.25 ± 6.15 kg). No statistical difference was observed between the two groups for parameters such as gender, age, weight, and operation type (*P* > .05). The screening of the participants and subsequent grouping are shown in Figure S1.

### 2.3 Anesthesia methods

All pediatric patients were fasted for 6 hours and prohibited to drink 4 hours prior to the operation. They were treated in a conventional manner with an intramuscular injection with 0.2 mg/kg midazolam (H10980025, Nhwa Pharma Co., Ltd., Nhwa Pharmaceutical Factory, Jiangsu, China) and 0.02 mg/kg atropine (H50020044, Xinan Pharmaceutical Chemical Co., Ltd.). The patients were transferred to the operating theaters upon sleeping, after which they received an intravenous infusion and routine nasal oxygen. The patients in the observation group were administered with an intravenous injection of 2 mg/kg ketamine (H14022824, Shanxi Taiyuan Pharmaceutical Chemical Co., Ltd.) and 2 mg/kg lidocaine (H20133209, Hubei Tianyao Pharmaceutical Chemical Co., Ltd.) at the ratio of 1:1. The patients in the control group only underwent an intravenous administration with 2 mg/kg ketamine. When tracheal intubation was required during the operation, it was conducted 3 minutes after administering the anesthetic injection, followed by a mechanical ventilation by connecting the machine, with the respective respiratory parameters: tidal volume of 10‐15 mL/kg, respiratory frequency of 16‐24 times/min, inspiration‐expiration ratio of 1:1.5, and partial pressure of end‐tidal carbon dioxide maintained at 35‐45 mm Hg. On the basis of the limb movements of pediatric patients and the length of operation time, anesthesia requirement was maintained by a fractional intravenous injection. During the operation, the anesthetic dose could be adjusted according to the anesthetic effect.

### 2.4 Observation of indices

Changes of systolic blood pressure (SBP), heart rate (HR), respiratory rate (RR) and blood oxygen saturation (SpO_2_) pre‐anesthesia, intra‐ and post‐operation were observed using a multifunctional monitor in a continuous manner. The levels of alanine aminotransferase (ALT), aspartate aminotransferase (AST), urea nitrogen (BUN), and creatinine (Cr) were also detected using an immunoturbidimetric assay (ITA) in the Au2700 automatic biochemical analyzer (Olympus Corporation). Following the operation, some secretions in the oropharynx were extracted. Then, the tracheal tube was removed after ensuring effortless breathing of the patients; the pharynx and larynx reflex recovered and could be further awakened. When the SpO_2_ concentration during spontaneous breathing reached above 0.98, the patients were sent to the care unit, to monitor the vital signs and oxygen inhalation for more than 1 hour by mask. Subsequently, the onset time and the duration time of anesthesia, ketamine dose, the recovery time, and the occurrence of various postoperative adverse reactions such as nausea and vomiting, suctioning, shiver, dysphoria and lethargy were documented. The patients with unobstructed breathe under the condition of no oxygen inhalation, SpO_2_ > 95%, conscious movement, and crying were regarded as the standard to determine whether there was substantial recovery or not in pediatric patients. The onset time of anesthesia was defined as follows: patients changed from a waking state to a sleeping state after an anesthetic injection, with simultaneous loss of consciousness and relaxation of the body muscles. The duration time of anesthesia was calculated from the onset of anesthesia to the reinjection of anesthetics. The time of recovery was defined as follows: after the operation, the concentration of anesthetics gradually decreased as the excretion of anesthetics from the patients’ body until patients could breathe freely and were conscious.

### 2.5 Anesthetic effect criteria

Through the course of the operation, no body movement was judged as excellent anesthetic effect; body movement having no influence on the operative procedure and no need for further anesthetic administration was assessed as good anesthetic effect; strong body movement interfering with the operation so as to increase the anesthetic dose was regarded as poor anesthetic effect.

### 2.6 Drug concentration monitoring and measurement

With drug induced pre‐anesthesia as a baseline, venous blood was collected for the patients prior to drug administration. Next, repeated venous blood collection was conducted for the pediatric patients after administration at 5, 15, 30, 60, 120, 240, and 480 minutes, respectively. The venous blood was allowed to stand for 30 minutes, and then centrifuged at 1610 *g* for 10 minutes, after which the serum was collected. High‐performance liquid chromatography (HPLC; Spectra‐Physics Analytical) was used to measure the ratio of ketamine/internal standard peak height, in which the detection wavelength was set at 215 nm, the injection volume was 60 μL at 1 mL/min, and the mobile phase was the mixture of acetonitrile, methanol, and monometallic sodium orthophosphate at a ratio of 32:51: 16. Standard curve *Y* = 1.0862*X* − 0.0072 was employed to calculate the value with ketamine/internal standard peak height ratio as *Y*‐axis and ketamine concentration as *X*‐axis; and the correlation coefficient “*r*” was .9995; the lowest limit of detection was 10 ng/L; and the recovery rates were 96% (0.5 g/mL, n = 2) and 92.3% (0.2 g/mL, n = 2). According to the changes of ketamine concentration, two groups of compartment models were fitted to two‐compartment model (Model 7) with the Winnolin software, and pharmacokinetic parameters were detected using a computer, and further assessed by the combination with Akaike information criterion.

### 2.7 Maze and decoding test

In the experiment, the pediatric patients were treated with maze and decoding test before and after the operation for evaluating cognition function. At first, a demonstration on a sample paper with a pencil was given to patients as an illustration; next, the patients were facilitated to depict the maze path on the test paper, followed by calculation of the correct numbers of the maze in quantitative time, which served as the value of maze. The patients underwent observation of a set of simple graphs and symbols (each graph had its symbol) and were asked to draw the corresponding symbol in response to the given graph. Right answer was credited as one score to the patient. The correct numbers of graphic matching by pediatric patients within 2 minutes were defined as the decoding value.

### 2.8 Statistical analysis

All statistical data analyses were conducted using the SPSS 20.0 software (IBM Corp.). The measurement data were expressed as mean ± standard deviations. Initially, normal distribution and homogeneity of variance were tested for all the data. If data conformed to normal distribution and homogeneity of variance, comparison between groups was analyzed by unpaired *t* test, data in different group were analyzed by one‐way analysis of variance (ANOVA), and pairwise comparisons were conducted using Tukey's post hoc test. The data with skew distribution or unequal variances were compared by the rank sum test. Categorical data were measured using a Chi‐square test, and rank data were compared with rank sum test. A value of *P* < .05 indicated statistical significance. As for one‐way ANOVA, the power observed was determined to be 80.53%, and for repeated measurement ANOVA, the power was determined to be 90.75%. This was based on the effect value of 0.8165 obtained by calculating the concentration of ketamine in serum at 5 minutes (peak value) and 240 minutes after administration. Power calculation was analyzed using the G* Power Software.

## 3 RESULTS

### 3.1 Baseline characteristics of patients

The observation group was comprised of 170 pediatric patients with hernia repair, 71 with cleft lip and palate repair, and 52 with appendectomy; 237 males and 56 females, aged between 1 and 12 years (mean age of 6.91 ± 2.23 years) and weighing 8.11‐45.17 kg (mean weight of 25.93 ± 6.92 kg), respectively. While the control group consisted of 168 cases with hernia repair, 69 cases with cleft lip and palate repair, and 56 cases with appendectomy. Among them, there were 241 males and 52 females, aged between 1 and 13 years (mean age of 6.63 ± 2.17 years) and weighing 8.04‐42.48 kg (mean weight of 25.25 ± 6.15 kg). No statistical difference was observed between the two groups for parameters such as gender, age, weight, and operation type (*P* > .05; Table 1).

**Table 1:** The baseline characteristics of patients administered with ketamine alone or with lidocaine

Characteristics	Observation	Control	*t*/*χ* ^2^	*P*
Gender (male/female)	237/56	241/52	0.182	.670
Age	6.91 ± 2.23	6.63 ± 2.17	1.54	.124
Weight	25.93 ± 6.92	25.25 ± 6.15	1.257	.209
Type of operation				
Hernia repair	170	168	0.189	.910
Cleft lip and palate repair	71	69		
Appendectomy	52	56		

### 3.2 Pediatric patients receiving intravenous injections of ketamine/lidocaine combination show stable vital signs during operation

As shown in Table 2, insignificant differences were evident in SBP, HR, and RR between the observation and control groups before anesthesia (*P* > .05). SBP, HR, and RR in the observation group were lower intra‐ and post‐operation compared with the control group (*P* < .05). SBP, HR, and RR of the control group were increased intra‐ and post‐operation compared with the values before administration (all *P* < .05), while the observation group exhibited no statistical differences (all *P* > .05), which indicated that the vital signs of pediatric patients in the observation group were stable. Between the two groups, SpO_2_ was maintained between 97% and 100% pre‐anesthesia, intra‐ and post‐operation (*P* > .05).

**Table 2:** SBP, HR, RR, and SpO_2_ levels of patients administered with ketamine alone or with lidocaine during and after operation

Index	Group	Pre‐anesthesia	*P*	Intra‐operation	*P*	Post‐operation	*P*
SBP (mmHg)	Observation	92.2 ± 17.0	.142	(−0.0081) ± 0.0008	<.001	(−0.0208) ± 0.0011	<.001
	Control	94.2 ± 16.9		0.3383 ± 0.0006*		0.2128 ± 0.0012*e	
HR (T/min)	Observation	91.3 ± 21.1	.763	(−0.0264) ± 0.0007	<.001	(−0.0237) ± 0.0008	<.001
	Control	91.8 ± 19.7		0.3313 ± 0.0009*		0.2367 ± 0.0008*	
RR (T/min)	Observation	20.9 ± 3.6	.06	(−0.0294) ± 0.0012	<.001	(−0.0128) ± 0.0007	<.001
	Control	22.8 ± 5.1		0.2581 ± 0.0013*		0.1512 ± 0.0009*	
SpO_2_ (%)	Observation	98.7 ± 0.9	.072	(−0.0061) ± 0.0011	.097	(−0.003) ± 0.0039	.07
	Control	98.9 ± 0.7		(−0.0059) ± 0.009		(−0.0036) ± 0.0041	

Note

The statistical values of the data obeying the normal distribution between the observation group and the control group were >0.05.

Abbreviations: HR, heart rate; RR, respiratory rate; SBP, systolic blood pressure; SpO_2,_ blood oxygen saturation.

* Compared with pre‐anesthesia, *P* < .05.

### 3.3 The safety is the same whether ketamine is administered alone or with lidocaine

In our findings, only the control group demonstrated a case with ALT changes of clinical significance (reached 152 μ/L), while the remaining cases were well within the normal range. No significant differences were evident in ALT, AST, BUN, and Cr before administration, as well as intra‐ and post‐operation between the observation group and the control group (all *P* > .05). Intra‐ and post‐operation, ALT, AST, BUN, and Cr were higher in both the observation group and the control group (all *P* < .05), while the rising degree of ALT, AST, BUN, and Cr in the control group was higher than those in the observation group without any statistical differences (all *P* > .05) (Table 3).

**Table 3:** ALT, AST, BUN, and Cr levels when ketamine is administered alone or with lidocaine at pre‐anesthesia, intra‐operation, and post‐operation

Index	Group	Pre‐anesthesia	*P*	Intra‐operation	*P*	Post‐operation	*P*
ALT (U/L)	Observation	17.1 ± 6.0	.782	0.29 ± 0.18*	.064	0.16 ± 0.09*	.254
	Control	17.3 ± 5.3		0.32 ± 0.21*		0.17 ± 0.12*	
AST (U/L)	Observation	23.1 ± 6.1	.56	0.25 ± 0.09*	.229	0.13 ± 0.07*	.108
	Control	22.8 ± 7.1		0.26 ± 0.11*		0.12 ± 0.08*	
BUN (mmol/L)	Observation	4.3 ± 1.5	.437	0.11 ± 0.08*	.07	0.09 ± 0.06*	.064
	Control	4.4 ± 1.3		0.12 ± 0.05*		0.10 ± 0.07*	
Cr (umol/L)	Observation	62.6 ± 16.8	.095	0.16 ± 0.07*	.134	0.10 ± 0.06*	.064
	Control	64.7 ± 14.5		0.17 ± 0.09*		0.09 ± 0.07*	

Note

The statistical values of the data obeying the normal distribution between the observation group and the control group were >0.05.

Abbreviations: ALT, alanine aminotransferase; AST, aspartate aminotransferase; BUN, urea nitrogen; Cr, creatinine.

* Compared with pre‐anesthesia, *P* < .05.

### 3.4 Ketamine/lidocaine combination reduces recovery agitation in pediatric patients

As shown in Table 4, a general dose of ketamine in the observation group was lower than that in the control group; the time of onset and duration of anesthesia, and postoperative recovery time were shorter in the observation group compared with the control group (all *P* < .05).

**Table 4:** General doses of ketamine, the time of onset and duration of anesthesia, and postoperative recovery time when ketamine is administered with lidocaine in pediatric patients during the operation

Group	Observation (n = 293)	Control (n = 293)	*P*
General ketamine dose (mg)	53.9 ± 11.8	80.7 ± 2.0	<.001
Onset time (min)	2.9 ± 0.5	4.3 ± 0.9	<.001
Duration time (min)	48.6 ± 7.2	61.3 ± 9.0	<.001
Recovery time (min)	6.8 ± 1.2	15.8 ± 4.9	<.001

### 3.5 Ketamine/lidocaine combination reduces the incidence of adverse reactions

As shown in Table 5, the excellent rate of anesthesia in the observation group was higher than the rate in the control group (*P* < .05). The incidence rate of various adverse reactions like nausea and vomiting, suctioning, shiver, dysphoria, and lethargy of anesthesia was lower in the observation group compared with the control group, with a lower number of overall adverse reactions in the observation group than the control group (all *P* < .05).

**Table 5:** Anesthesia effects and adverse reactions when ketamine is administered alone or with lidocaine during the operation

Group	Observation (n = 293)	Control (n = 293)	*x* ^2^	*P*
Anesthesia effects				
Excellent	187 (63.8%)	99 (33.8%)	18.38	<.001
Good	70 (23.9%)	63 (21.5%)	0.30	.584
Poor	36 (12.3%)	131 (44.7%)	42.74	<.001
Adverse reactions	57	194	53.79	<.001
Nausea and vomiting	24 (8.2%)	59 (20.1%)	12.96	<.001
Suctioning	8 (2.7%)	35 (12.0%)	15.82	<.001
Shiver	5 (1.7%)	28 (9.6%)	15.20e	<.001
Dysphoria	9 (3.1%)	38 (13.0%)	16.60	<.001
Lethargy	11 (3.8%)	34 (11.6%)	10.93	<.001

### 3.6 Ketamine/lidocaine combination maintains plasma concentrations of ketamine, reduces ketamine clearance, and prolongs its half‐life

**Figure 1:**
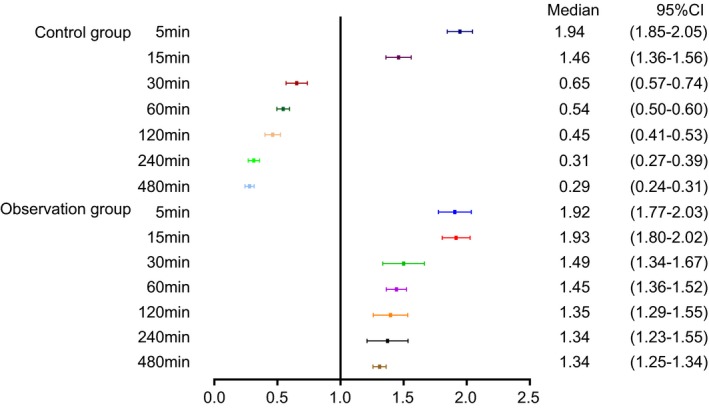
The HPLC results suggest that ketamine combined with lidocaine could maintain plasma concentrations of ketamine, reduce ketamine clearance, and prolong its half‐life. ^*^Compared with the control group, *P* < .05 (data were presented in median [95% confidence interval])

Ketamine concentrations of pediatric patients at each time point in the observation and the control groups are shown in Figure 1 (Blood drug concentration = [values detected at different time points ‐ baseline values]/baseline values). In comparison with the control group, before and after administration at 5, 15, and 480 minutes, no statistical differences were evident in the concentration of ketamine in the observation group (*P* > .05), but it was higher than the value in the control group at 30, 60, 120, and 240 minutes (*P* < .05). As shown in Table 6, compared with the control group, the elimination half‐life of ketamine in the observation group was prolonged, the AUC was increased, and the plasma clearance rate was decreased (all *P* < .05).

**Table 6:** Comparison of pharmacokinetic parameters when ketamine is administered alone or with lidocaine at 30, 60, 120, and 240 min after administration in pediatric patients

Group	Observation (n = 293)	Control (n = 293)	*P*
Distribution half‐life (min)	13.7 ± 1.4	13.5 ± 1.1	.092
Elimination half‐life (min)	262.9 ± 15.1	164.2 ± 10.0	<.001
VDSS (L/kg)	1.9 ± 0.6	2.0 ± 0.5	.071
AUC [mg/(min^*^mL)]	278.0 ± 26.1	163.9 ± 14.0	<.001
Plasma clearance rate [mg/(kg^*^min)]	7.3 ± 0.6	13.9 ± 0.9	<.001

Abbreviations: AUC, area under the curve; VDSS, volume of distribution at stead state.

### 3.7 Ketamine/lidocaine combination favors postoperative cognition function

The scores of maze and decoding test among the observation and control groups are shown in Table 7, which exhibited no significant differences between the two groups before administration (both *P* > .05). In the observation group, the scores of maze and decoding test post‐operation were lower than that during pre‐anesthesia, with no significant differences (both *P* > .05); however, those in the control group were decreased compared with the same pre‐anesthesia (both *P* < .05). The scores in the observation group were higher than the scores observed in the control group (both *P* < .05).

**Table 7:** Comparison of cognition function before and after anesthesia when ketamine is administered alone or with lidocaine

Group	Time	Maze test (score)	*P*	Decoding (score)	*P*
Observation	Pre‐anesthesia	6.1 ± 2.0	.055	41.0 ± 7.0	0.068
	Post‐operation	5.8 ± 1.4		39.7 ± 10.1	
Control	Pre‐anesthesia	6.1 ± 0.8	<.001	41.3 ± 3.5	<0.001
	Post‐operation	4.1 ± 0.8*		30.0 ± 5.5*	

* Compared with the observation group post‐operation, *P* < .05.

## 4 DISCUSSION

Surgical care of all patients relies on proper anesthesia.^15^ Anesthetic exposure may work as a specific marker of ascended risk,^16^ irrespective of risk association with patient characteristics.^2^ Principally in young children, the administration of general anesthesia should be performed if it emerges as a necessity and the general anesthesia duration should be as shorter as well.^16^ With an effort to improve the current anesthetic practice, this study was performed to explore the efficacy and safety of ketamine use combined with lidocaine on pediatric anesthesia.

Ketamine has been extensively used in the emergency department for various emergencies including conscious sedation and rapid sequence induction.^17^ Ketamine inhibits morphine metabolism to increase the duration of analgesia, thereby exercising its intrinsic anti‐inflammatory effects.^18^, ^19^ A report by Hwang et al^20^ revealed that ketamine could potentially induce sympathetic stimulation, accompanied by an increase in BP and HR. Moreover, as a local anesthetic, lidocaine is a member of the amide group.^21^ Existing literature has signified its systemic use for analgesic effects in various human experimental studies and animal pain models.^22^, ^23^ Lidocaine confers cerebral protection and could improve the cognitive performance of elderly patients who underwent spine surgery.^24^

In our study, the findings exhibited the combination of ketamine and lidocaine to be more effective and safer for pediatric anesthesia, which was evidenced by more stable vital signs (reduced SBP, HR, and RR) of pediatric patients in response to the combination treatment with ketamine and lidocaine than ketamine treatment alone. It has been suggested that IV lidocaine could potentiate changes in HR by interacting with sodium channels and repressing cellular calcium influx, thus inducing changes in depolarization and conduction velocity in myocardial Purkinje fibers.^25^ SBP change may be observed on an arterial pressure waveform induced by changes in intrathoracic pressure resulting from controlled ventilation.^26^ Consistent with our study, Kaka et al^27^ reported that the combination of ketamine with lidocaine potentially enhances the anesthetic effects, reduces drug‐related side effects, and decreases the opioid requirement and postoperative adverse reactions. As previously reported, combination of lidocaine and ketamine was predominantly effective in 73% of patients with acute neuropathic pain.^28^ A combination of ketamine with promethazine is effective in the sedation of pediatric dental patients by eliminating the incidence of vomiting and enhancing the sedative efficacy.^29^

Additionally, a lower dose of general ketamine was evident in the observation group than the control group; the time of onset and duration of anesthesia, and postoperative recovery time were shorter than those in the control group. A conjoint administration of lidocaine with ketamine shortens the onset of anesthesia in mice and improves the anesthetic efficacy without prolonging the recovery time.^30^, ^31^ Besides, the addition of ketamine to epidural lidocaine or bupivacaine as an additive anesthetic has been identified to increase the duration of regional anesthesia and postoperative analgesia, and peri‐incisional use of 0.3%‐0.5% ketamine combined with local anesthetic in surgical wounds could enhance analgesia.^32^ Pretreatment with a combination of intravenous lidocaine 40 mg and ketamine 25 mg is a more effective anesthetic than lidocaine 40 mg or ketamine 25 mg alone in preventing subsequent pain.^20^ Reports have illustrated that there is no interaction between ketamine and intravenous lidocaine when administered at antihyperalgesic/low doses.^33^ Lidocaine is a kind of amides commonly used in clinical local anesthesia, which has good analgesic and local anesthetic effects. The specific analgesic effect increases with the increase of dose of lidocaine, but there are more toxic and side effects when lidocaine is excessively applied. Ketamine mainly plays an anesthetic effect by inhibiting excitatory neurotransmitters and interacting with N‐methyl‐d aspartate and improves the comfort of patients during and after operation. Since ketamine has obvious excitatory effect on cardiovascular system, while lidocaine has a slight inhibitory effect on circulation, the combination of the two can make the hemodynamics more stable during operation.^11^, ^33^, ^34^

Additionally, the incidence rate of adverse reactions after anesthesia in the observation group was lower than the rate in the control group. Existing reports have documented the appearance of less adverse reactions with ketamine used in children compared to those treated with midazolam and have ascertained lidocaine infusion to be safe with no severe side effects.^9^, ^35^ Patients receiving conjoint administration with ketamine and lidocaine exhibited significant clinical improvement in aneurysmal subarachnoid hemorrhage without any adverse reactions.^36^ Pharmacological agents have been demonstrated to attenuate the hemodynamic (orthosympathetic/stress) response to tracheal intubation in pediatric patients undergoing elective surgery, thus preventing a wide array of side effects.^37^ Besides, decreased general anesthesia can induce adverse laryngeal effects after operation, such as hoarseness and vocal cord injuries.^38^ After intravenous injection, lidocaine could not only reduce the adverse effects such as the increase of BP, HR, and myocardial oxygen consumption caused by the increase of catecholamine level induced by ketamine, but also diminish the increase of intracranial pressure and brain oxygen consumption induced by ketamine, thus effectively protect the brain tissue.^11^, ^33^, ^34^

In conclusion, the gathered evidence in our study provided information supporting the use of the combination of ketamine and lidocaine contributed to more stable vital signs, shorter onset and recovery time, prolonged elimination half‐life, increased area under curve, decreased plasma clearance rate, and less general dosage and adverse reactions. In this study, however, only two kinds of regimens were administered: ketamine and ketamine combined with lidocaine. Further studies are warranted in the future to determine the efficacy of multiple anesthetic agents such as morphine or fentanyl in comparison.

## AUTHOR CONTRIBUTIONS

HF involved in conception and design of research; HFL and PL performed the experiments; HFL, MY, and FXZ analyzed the data; RL and PCZ interpreted the results of experiments; RRW and QYW prepared the figures; HF and PCZ drafted the manuscript; HF and JPZ edited and revised the manuscript; HF, HFL, MY, FXZ, RL, RRW, QYW, PCZ, and JPZ approved final version of the manuscript.

## ETHICAL APPROVAL

The present study was conducted under the approval of the medical ethics committee of the Guizhou Provincial People's Hospital and Guizhou University People's Hospital. The participants and patient guardians included in the current study signed written informed consents.
